# *In Vivo* Injection of Anti-LGI1 Antibodies into the Rodent M1 Cortex and Hippocampus Is Ineffective in Inducing Seizures

**DOI:** 10.1523/ENEURO.0267-22.2023

**Published:** 2023-03-13

**Authors:** Paul Baudin, Delphine Roussel, Séverine Mahon, Stéphane Charpier, Vincent Navarro

**Affiliations:** 1 Sorbonne Université, Institut du Cerveau - Paris Brain Institute - ICM, Inserm, CNRS, APHP, Hôpital de la Pitié-Salpêtriére, 75013 Paris, France; 2 AP-HP, Hôpital de la Pitié-Salpêtriére, DMU Neurosciences 6, Epilepsy Unit and Clinical Neurophysiology Department, 75013 Paris, France; 3 Center of Reference for Rare Epilepsies, APHP, Hôpital de la Pitié-Salpêtrière, 47 Boulevard de l’Hôpital, 75013 Paris, France

**Keywords:** autoimmune encephalitis, electrophysiology, epilepsy, LGI1, video-EEG

## Abstract

Autoimmune encephalitis (AIE) associated with antibodies directed against the leucine-rich glioma inactivated 1 (LGI1) protein is the second most common AIE and is responsible for deleterious neocortical and limbic epileptic seizures. Previous studies demonstrated a pathogenic role of anti-LGI1 antibodies via alterations in the expression and function of Kv1 channels and AMPA receptors. However, the causal link between antibodies and epileptic seizures has never been demonstrated. Here, we attempted to determine the role of human anti-LGI1 autoantibodies in the genesis of seizures by analyzing the impact of their intracerebral injection in rodents. Acute and chronic injections were performed in rats and mice in the hippocampus and primary motor cortex, the two main brain regions affected by the disease. Acute infusion of CSF or serum IgG of anti-LGI1 AIE patients did not lead to the emergence of epileptic activities, as assessed by multisite electrophysiological recordings over a 10 h period after injection. A chronic 14 d injection, coupled with continuous video-EEG monitoring, was not more effective. Overall, these results demonstrate that acute and chronic injections of CSF or purified IgG from LGI1 patients are not able to generate epileptic activity by themselves in the different animal models tested.

## Significance Statement

Anti-leucine-rich glioma inactivated 1 (LGI1) encephalitis is a frequent and severe autoimmune encephalitis. Several previous studies have shown a pathogenic role of anti-LGI1 antibodies, but their link with the emergence of seizures has never been demonstrated. To study the role of anti-LGI1 autoantibodies in the genesis of seizures, we performed acute and chronic injections of CSF and purified serum IgG of anti-LGI1 encephalitis patients in rodents, targeting the two main brain regions affected by the disease, the hippocampus and primary motor cortex. Brain activities were monitored for 10 h after acute injections, and for 1 month after the beginning of chronic injections. Our results show that chronic and acute injections of anti-LGI1 antibodies were ineffective in inducing epileptic activity in rats and mice.

## Introduction

Autoimmune encephalitis (AIE) is a group of autoimmune syndromes that are responsible for an increasing number of unexplained drug-resistant epilepsies (Dalmau et al., 2017; Goodfellow and Mackay, 2019; Husari and Dubey, 2019). The second most frequent AIE, which is caused by the production of antibodies directed against the leucine-rich glioma inactivated 1 protein (anti-LGI1 AIE), is associated with limbic encephalitis and tonic–dystonic motor seizures (TDSs). The evolution of the disease, generally within months, is characterized by a progressive bilateral involvement of limbic and motor brain structures (Navarro et al., 2016) and a gradual increase in seizures frequency, possibly leading to life-threatening status epilepticus. An absence or delay in diagnosis and treatment can thus have devastating consequences and lead to irreversible lesions, such as hippocampal atrophy (Ghimire et al., 2020; Griffith et al., 2020; Liu et al., 2020; Ramanathan et al., 2021).

LGI1 is a 60 kDa secreted glycoprotein, ubiquitously expressed in the CNS. It takes part in a trans-synaptic complex, including Kv1.1 voltage-dependent potassium channels and glutamatergic AMPA receptors (AMPARs; Schulte et al., 2006; Yamagata et al., 2018; Fukata et al., 2021). LGI1 is also enriched at the axon initial segment, where it colocalizes with Kv1.1 channels (Seagar et al., 2017; Hivert et al., 2019). LGI1 is highly conserved between human and rodents. Previous studies reported a specific labeling of human anti-LGI1 IgG on rodent wild-type hippocampal slices, suggesting that the human autoantibodies effectively react with the rodent protein (Kornau et al., 2020; Ramberger et al., 2020; Extrémet et al., 2022). Anti-LGI1 antibodies have been shown to prevent fixation of LGI1 to its presynaptic and postsynaptic partners or to induce the internalization of the LGI1-associated protein complex (Ramberger et al., 2020), resulting in alterations of the expression and functioning of Kv1.1 channels and AMPAR (Ohkawa et al., 2013; Petit-Pedrol et al., 2018; Kornau et al., 2020; Ramberger et al., 2020; Extrémet et al., 2022).

However, the pathophysiological mechanisms linking these abnormal molecular interactions to the emergence of seizures remain unclear. Several groups have attempted to develop *in vivo* animal models of anti-LGI1 AIE, especially through the passive transfer into the murine brain of total Ig or purified anti-LGI1 antibodies from patients. They observed a decrease in Kv1.1 and AMPAR expression, but none of these previous studies used electrophysiological measurements to assess the occurrence of seizure activities in the involved brain structures of interest (Petit-Pedrol et al., 2018; Ramberger et al., 2020).

Here, we sought to characterize in rodents the functional impact of intracerebral injections of anti-LGI1 antibodies from patients. Antibodies from either CSF or patient serum were injected into the hippocampus or primary motor cortex (M1), the two main target regions of the human disease (Navarro et al., 2016). Because action kinetics of autoantibodies are not known, we set up protocols to study its acute and chronic impacts. To avoid species-dependent effects, the effects of intracerebral injections were examined in both rats and mice.

## Materials and Methods

### Patients

CSF and serum of patients were obtained from the Department of Neurology at the Pitié-Salpêtrière Hospital. The main patients’ characteristics are summarized in Table 1. In anti-LGI1 AIE (LGI1 patients, *n *=* *3), serum and CSF were collected before the start of immunotherapy, and the presence of anti-LGI1 antibodies was assessed on HEK293 cells expressing LGI1 (catalog #FA1439-1003-1, EuroImmun). The group of control patients (*n *=* *3) included one nonepileptic patient with a negative autoantibody test, who had been hospitalized for repeated discomfort of psychogenic origin. The two other control subjects were LGI1 patients, who were sampled 1 year after the complete remission of their AIE. These two patients were free of neurologic symptoms and tested negative for antibodies in serum and CSF. None of the control patients had neurodegenerative diseases, brain tumor, or acute inflammatory pathology. Their CSF was normal in terms of cellularity, proteinorachia, and glycorrhachia. Samples from different patients were used independently to be able to assess any specific patient-dependent effect and to avoid drawing general conclusions from effects caused by a specific patient profile. The protocol was sponsored by INSERM (Agreement #C16-16, 20152482) and was approved by a local ethics committee.

**Table 1:** Main characteristics of the included patients

	LGI1-1	LGI1-2	LGI1-3	Ctrl-1	Ctrl-2	Ctrl-3
Age (years)	21	65	75	20	22	67
Sex	F	M	M	M	F	M
Inclusion criteria	Anti-LGI1 Abs in serum and CSF	Anti-LGI1 Abs in serum and CSF	Anti-LGI1 Abs in serum and CSF	Repeated discomfort of nonepileptic origin	Patient LGI1-1 after remission	Patient LGI-2 after remission
TDS (*n/*h)	Yes (17)	Yes (23)	Yes (7)	No	No	No
Mesial temporal seizures	Yes	No	No	No	No	No
Hyponatremia	Yes	No	No	No	No	No
CSF (cellularity, proteinorachia, glycorachia)	Normal	Normal	Normal	Normal	Normal	Normal
Serum anti-LGI1/Ab titration ratio	1:32	1:64	1:32			

Three patients were included in the LGI1 group (LGI1-1, LGI1-2, and LGI1-3), and three in the control group (ctrl-1, ctrl-2, and ctrl-3). The serum anti-LGI1 Ab titration ratio corresponds to the last dilution of the samples in which a staining on HEK cells was detected. Ab, Antibody; Ctrl, control; F, female; M, male.

### Purification of IgG from patient serum

Total IgG was extracted from the patients’ serum and purified. IgGs were isolated using protein A columns (PURE1A-KIT, Sigma-Aldrich). After IgG isolation, samples were dialyzed, normalized to a concentration of 1 mg/ml in PBS using Amicon 30 kDa ultrafiltration filters (catalog #UFC503096, Sigma-Aldrich), filtered, and stored at −80°C until use. The presence of anti-LGI1 antibodies was verified after purification by labeling on HEK293 cells expressing LGI1, revealed by fluorescent human anti-IgG (catalog #FA1439-1003-1, EuroImmun). Semiquantitative estimation of anti-LGI1 antibodies was performed on the IgG purified from serum samples, through titration and labeling on HEK293 cells expressing LGI1 (catalog #FA1439-1003-1, EuroImmun). We diluted the IgG sample progressively by adding PBS such as the volume was multiplied by two between each staining. The titration ratio for each sample is reported in Table 1 and corresponds to the last dilution in which we could detect a staining on HEK cells.

### Animals

Experiments were performed on 61 Sprague Dawley male rats and 27 C57BL/6J mice, all between 8 and 16 weeks of age (Charles River Laboratories). The C57BL/6J mouse strain was chosen as it is commonly used in previous *in vitro* and *in vivo* studies addressing the effects of anti-LGI1 human autoantibodies (Petit-Pedrol et al., 2018; Kornau et al., 2020; Ramberger et al., 2020). The Sprague Dawley rat strain is the one used to develop our LGI1 encephalitis seizure-like model, which recapitulates the electrical and behavioral hallmarks of patients (Baudin et al., 2022). Both rats and mice were used to verify that the absence of seizure was not species specific.

The experiments detailed below complied with the European Union guidelines (Directive 2010/63/EU) and were approved by the French Ministry of Research, and the local Ethics Committee. The number of animals used in each experimental group is detailed in Tables 2 and 3.

**Table 2:** Experimental configurations for acute injection of CSF or purified total IgG

	LGI1 CSF	Control CSF	LGI1 patient-derived serum IgG	Control patient-derived serum IgG
M1	LGI1-1: *n *=* *3	Ctrl-1: *n *=* *2	LGI1-1: *n *=* *2	Ctrl-1: *n *=* *1
	LGI1-2: *n *=* *2	Ctrl-2: *n *=* *1	LGI1-2: *n *=* *2	Ctrl-2: *n *=* *1
	LGI1-3: *n *=* *3	Ctrl-3: *n *=* *2	LGI1-3: *n *=* *2	Ctrl-3: *n *=* *1
Hippocampus	LGI1-1: *n *=* *2	Ctrl-1: *n *=* *2	LGI1-1: *n *=* *2	Ctrl-1: *n *=* *1
	LGI1-2: *n *=* *2	Ctrl-2: *n *=* *1	LGI1-2: *n *=* *2	Ctrl-2: *n *=* *1
	LGI1-3: *n *=* *2	Ctrl-3: *n *=* *2	LGI1-3: *n *=* *2	Ctrl-3: *n *=* *1

All these experiments were performed on rats.

**Table 3:** Experimental configurations for chronic hippocampal injection of CSF or purified total IgG in rats and mice

	LGI1 CSF	Control CSF	LGI1 patient-derived serum IgG	Control patient-derived serum IgG
Mice	LGI1-1: *n *=* *2	Ctrl-1: *n *=* *2	LGI1-1: *n *=* *3	Ctrl-1: *n *=* *2
	LGI1-2: *n *=* *2	Ctrl-2: *n *=* *2	LGI1-2: *n *=* *3	Ctrl-2: *n *=* *1
	LGI1-3: *n *=* *2	Ctrl-3: *n *=* *2	LGI1-3: *n *=* *2	Ctrl-3: *n *=* *1
Rat	LGI1-1: *n *=* *2	Ctrl-1: *n *=* *2	NA	NA
	LGI1-2: *n *=* *2	Ctrl-2: *n *=* *2		
	LGI1-3: *n *=* *2	Ctrl-3: *n *=* *2		

NA, Not available.

### Acute injections and multiscale *in vivo* recordings in sedated rats

**Figure 1.**
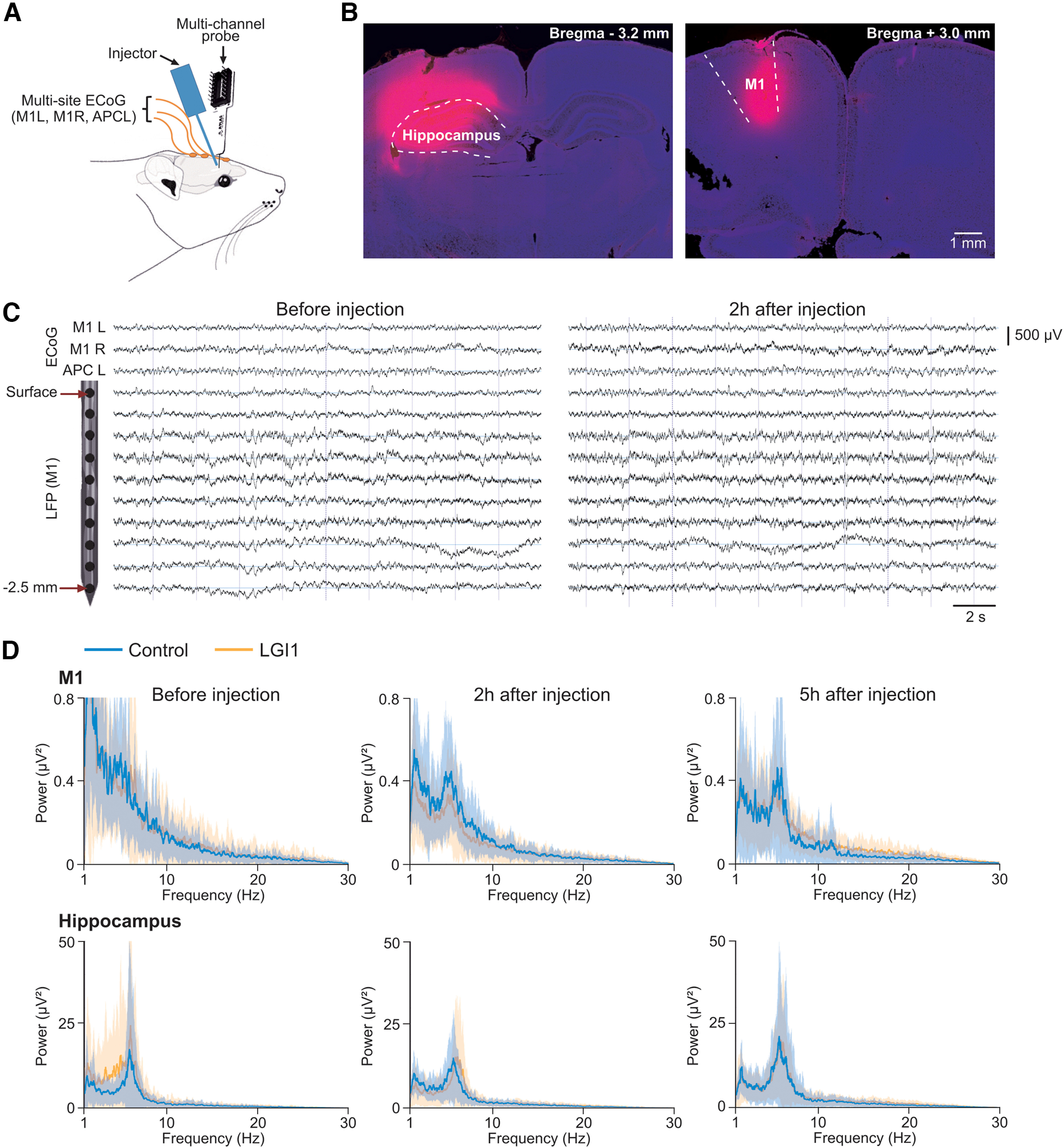
Acute injections of CSF and purified serum IgG. ***A***, Schematic of the experimental setup for acute injection and simultaneous electrophysiological recording from the sedated rat. ***B***, Control experiments for the location of the injection sites. Coronal brain slices at the indicated coordinates, showing the hippocampal (top) and M1 cortical (bottom) injection sites, as revealed by Fluoro-Ruby injections (pink), at the exact same volume and coordinates as the control and anti-LGI1 antibody-containing CSF and purified serum injected. Blue labeling is DAPI labeling of cell nuclei. ***C***, ECoG activities and local field potentials recorded before (left) and after (right) an acute injection of purified IgG from LGI1 patients into the M1 cortex. ECoG activity was collected from the left M1 (M1L), right M1 (M1R), and the left APC (APCL). A multichannel electrode was inserted in M1L, 200 µm anterior to the injection site to record LFPs from the different cortical layers. Note the absence of epileptiform activity or seizures on the postinjection recordings. ***D***, Frequency power (mean ± SD) of intracerebral LFP recordings after injection in M1 (top) and hippocampus (bottom), in control (blue) and LGI1 (orange) conditions. Electrophysiological signals were analyzed before injection (left), 2 h after injection (middle), and 5 h after injection (right). The electrode closest to the injection site was selected for the analysis. No significant difference was found between control (M1, *n *=* *8; hippocampus, *n *=* *8) and LGI1 (M1, *n *=* *14; hippocampus, *n *=* *12) experiments (two-tailed Mann–Whitney rank-sum test for each time period, on data binned in frequency bands of 1 Hz width). The statistical power estimated for each frequency bin was on average 0.55 ± 0.15. The frequency content of LFP activities is displayed separately between CSF and serum experiments in the Extended Data Figure 1-1. Stereotaxic coordinates used for the acute experiments are reported in the Extended Data Table 1-1.

Rats were first anesthetized by inhalation of 4% isoflurane (Osalia) and maintained with 2% isoflurane throughout the surgery. Animals were intubated to perform artificial ventilation (room air, 80 cycles/min, 2.6 ml/cycle) and placed on a stereotaxic frame. The incision and pressure areas were regularly infiltrated with lidocaine (2%; Centravet). CSF or purified patient IgG was injected using a Hamilton 1701 syringe with a 200-µm-outer diameter needle at a rate of 0.1 µl/min. Volumes of 0.5 and 2 µl were, respectively, injected into the left M1 and the left hippocampus. The localization of the injection sites was validated by injecting the fluorescent tetramethylrhodamine dextran-amine Fluoro-Ruby (Thermo Fisher Scientific) into the hippocampus (*n *=* *2) and M1 (*n *=* *2; Fig. 1*B*). Unilateral injections in the hippocampus or cortex were preferred to bilateral injections to avoid traumatizing large brain volumes and to offer the possibility to detect focal seizures and determine their initiation site.

10.1523/ENEURO.0267-22.2023.f1-1 Figure 1-1 ***A***, ***B***, Frequency content of LFP activities after acute injection of CSF (***A***) and serum IgG (***B***) from LGI1 and control patients in rat. ***A***, Frequency power (mean ± SD) of intracerebral LFP recordings after CSF injection in M1 (top) and hippocampus (bottom), in control (blue) and LGI1 (orange) conditions. Electrophysiological signals were analyzed before injection (left), 2 h after injection (middle), and 5 h after injection (right). The electrode closest to the injection site was selected for the analysis. No significant difference was found between control (M1, *n *=* *5; hippocampus, *n *=* *5) and LGI1 (M1, *n *=* *8; hippocampus, *n *=* *6) experiments (two-tailed Mann–Whitney rank-sum test for each time period, on data binned in frequency bands of 1 Hz width). ***B***, Same analysis as the one presented in ***A***, but after serum IgG injection. No significant difference was found between control (M1, *n *=* *3; hippocampus, *n *=* *3) and LGI1 (M1, *n *=* *6; hippocampus, *n *=* *6) experiments (two-tailed Mann–Whitney rank-sum test for each time period, on data binned in frequency bands of 1 Hz width). Download Figure 1-1, TIF file.

10.1523/ENEURO.0267-22.2023.tab1-1 Table 1-1 Coordinates used for injection and electrode placement in the acute injection experiments. All coordinates are expressed in millimeters from bregma, and were derived and adjusted from the rat brain atlas of Paxinos and Watson (1997a). Download Table 1-1, DOC file.

Multisite electrocorticographic (ECoG) recordings were made using low-impedance (60 kΩ) silver electrodes placed on the dura over the right M1, left M1, and left associative parietal cortex (APC). A reference electrode was apposed to the right temporal muscle. ECoG signals were amplified with a differential AC amplifier (model 1700, A-M Systems), filtered between 0.1 Hz and 1 kHz, and digitized at 3 kHz (model 1401 Micro3/Spike2 software version 7.20, Cambridge Electronic Design). Local field potentials (LFPs) were recorded using a multichannel linear probe of 16 electrodes separated by 250 µm (diameter, 35 µm; IrOx, ATLAS Neuro) inserted into M1 or in the hippocampus, 200 µm in front of the injection site. LFP signals were amplified and filtered between 0.1 Hz and 1 kHz, using a Digital Lynx amplifier (NeuraLynx), and digitized at 3.2 kHz. The stereotaxic coordinates for injection and recording sites are reported in the Extended Data Table 1-1. All coordinates were derived and adjusted from the rat brain atlas (Paxinos and Watson, 1997a). The experimental setup is schematically illustrated in Figure 1*A*.

After completion of surgery, isoflurane was gradually discontinued, and the animal was maintained in a sedated and analgesic state by repeated injections of sufentanil (3 µg/kg, i.p., every 30 min; Piramal), combined with repeated intramuscular injections of gallamine triethiodide (40 mg/2 h; Sigma-Aldrich) to allow for stable long-term recordings. Sufentanil sedation was chosen because it does not alter spontaneous activity and excitability of cortical cells (Altwegg-Boussac et al., 2014) and does not interfere with the induction and expression of focal and generalized seizures (Langlois et al., 2010; Depaulis et al., 2016). Heart rate and ECoG activity were continuously monitored to assess the depth of sedation. The stability of physiological parameters [i.e., end-tidal carbon dioxide concentration, oxygen saturation, and body temperature (37°C)], was verified throughout the experiments (Altwegg-Boussac et al., 2017). At the end of the experiments, animals were killed by injection of a lethal dose of euthasol (0.6 ml/kg; TVM). Their brains were then extracted to check the position of the injection syringes and intracranial recording probes. Briefly, after fixation with 4% paraformaldehyde, brains were frozen with isopentane, cut in 20 µm cryostat sections and stained with safranine (RAL Diagnostics; Williams et al., 2016).

### Chronic injection and long-term video-EEG recordings in freely moving rodents

Rats and mice were initially anesthetized by inhalation of 4% isoflurane and placed on a stereotaxic frame to implant chronic EEG electrodes and an intracerebral cannula. Analgesia, induced by subcutaneous injection of buprenorphine (0.05 mg/kg; Centravet), was initiated 40 min before isoflurane anesthesia. Anesthesia was maintained with 2% isoflurane to complete the surgical procedures, and body temperature was stabilized at 37°C with a homoeothermic blanket.

EEG recordings in M1 and APC were performed using 200-μm-diameter insulated stainless steel wire (A-M Systems). For rats, a small screw was welded to the stainless steel wires (catalog #19010–00, Fine Science Tools). In addition, an interlaced bipolar insulated stainless steel electrode was bilaterally inserted into the hippocampus. All electrodes were soldered to a six-pin female connector. Stereotaxic coordinates are reported in Extended Data Table 2-1. All coordinates were derived and adjusted from rat and mouse brain atlases (Paxinos and Watson, 1997a, b). An osmotic pump (model 1002, ALZET) was placed subcutaneously on the right flank of the animal and connected to an injection cannula (Brain Infusion Kit, ALZET) implanted in the right hippocampus to allow for a continuous injection of 84 µl for 14 d, at a flow rate of 0.25 µl/h. A reference electrode was placed over the cerebellum. Electrodes and cannula were fixed with surgical glue (Surgibond) and immobilized in dental cement on the skull of the animal.

After a recovery period of 3 d, implanted rats were placed under freely moving conditions in transparent custom-made recording cages, with *ad libitum* access to food and water. Continuous recordings of 24 or 48 h were performed every week with a complete video-EEG acquisition system. EEGs were amplified and digitized (Brainbox EEG-1166, Natus) at a sampling rate of 4096 Hz, filtered between 0.1 and 300 Hz. Video was synchronized to the electrophysiological signal and recorded at 25 frames/s. At the end of the recording period, animals were killed by injection of a lethal dose of euthasol (0.6 ml/kg; TVM). Brains were then removed and processed following the histologic procedures described above to check the position of the electrodes and injection cannula.

The IgG diffusion within the hippocampus was assessed in three mice and three rats, killed 7 d after surgery, after the infusion of purified IgG samples. Labeling was performed with biotinylated anti-human-IgG antibodies (catalog #BA-3000, Vector Laboratories), followed by enzymatic revelation.

### Analysis of electrophysiological signals

A visual reading of the recordings over a sliding window of 20 s was performed to evaluate the putative epileptic phenotype of rats and mice injected with human CSF and serum-purified IgG. Detection of epileptiform discharges or seizures was based on different criteria: an abrupt onset and termination, an amplitude threshold clearly different from the baseline activity (at least three times the SD), and/or abnormal changes in the background rhythm. In chronic recordings from freely moving animals, myoclonus and other epileptic movements, whether or not associated with events on the EEG, were also looked for on video recordings. For acute injections in sedated rats, a 30 min period was recorded before the injection. This served as a baseline against which the postinjection recordings were compared.

In acute experiments, a 15 min window was selected before injection, as well as 2 h and 5 h after injection. Fast Fourier transforms were computed on those windows, between 1 and 30 Hz. Power spectra were then binned in 1 Hz frequency bands, and compared between control and LGI1 experiments with a two-tailed Mann–Whitney rank-sum test. This analysis was performed with a combination of Fieldtrip (release 20200919; Oostenveld et al., 2011) and custom-developed scripts in MATLAB (version R2021b; MathWorks). M1 injection data and hippocampal injection data were analyzed independently. The statistical power was estimated for each frequency bin as the probability to find a difference of ±50% knowing the mean and SD control values, using the “sampsizepwr” built-in MATLAB function.

## Results

### Absence of seizure activity after acute intracerebral injection of CSF and purified serum IgG from LGI1 patients

To investigate the effect of anti-LGI1 antibodies on brain activity, we performed acute injections of CSF or purified serum IgG with anti-LGI1 antibodies into the hippocampus and M1 cortex of sedated rats, together with ECoG and LFP recordings (Fig. 1*A*,*B*). The different experimental configurations (*n *=* *42) are detailed in Table 2.

Baseline ECoG activity was recorded for 30 min before injection. The ECoG profile appeared desynchronized and dominated by relatively fast, small-amplitude cortical waves (Fig. 1*C*, left), as classically observed under sufentanil sedation (Altwegg-Boussac et al., 2014). Recording duration after injection varied between 5 and 11 h, with an average (±SD) of 7.2 ± 2.2 h (*n *=* *42 experiments). We first verified that the injection of CSF (*n *=* *14 experiments) and purified serum IgG (*n *=* *6 experiments) from control patients did not modify the background ECoG activity (data not shown). Given that the production of anti-LGI1 antibodies causes characteristic limbic and motor seizures in patients, we searched for a possible induction of epileptic seizures in experiments wherein anti-LGI1 antibodies containing CSF or serum-purified IgG were injected into the hippocampus (*n *=* *12) or M1 cortex (*n *=* *14). As illustrated in Figure 1*C* for an experiment in which recordings were performed in M1 before and after an injection of purified IgG serum at the same cortical site, we did not detect seizure-like events or interictal activity at the different ECoG locations. Epileptic activity was also absent from LFP recordings (Fig. 1*C*), regardless of the injection site. The frequency content of LFP activities after injection was similar between control and LGI1 experiments, when all experiments were pooled together (Fig. 1*C*,*D*) as well as when serum IgG experiments and CSF experiments were analyzed separately (Extended Data Fig. 1-1). CSF (*n *=* *24) and purified IgG serum (*n *=* *18) from LGI1 patients were similarly ineffective in inducing seizures.

### Absence of seizures after chronic intracerebral injections of CSF and purified serum IgG from LGI1 patients

**Figure 2.**
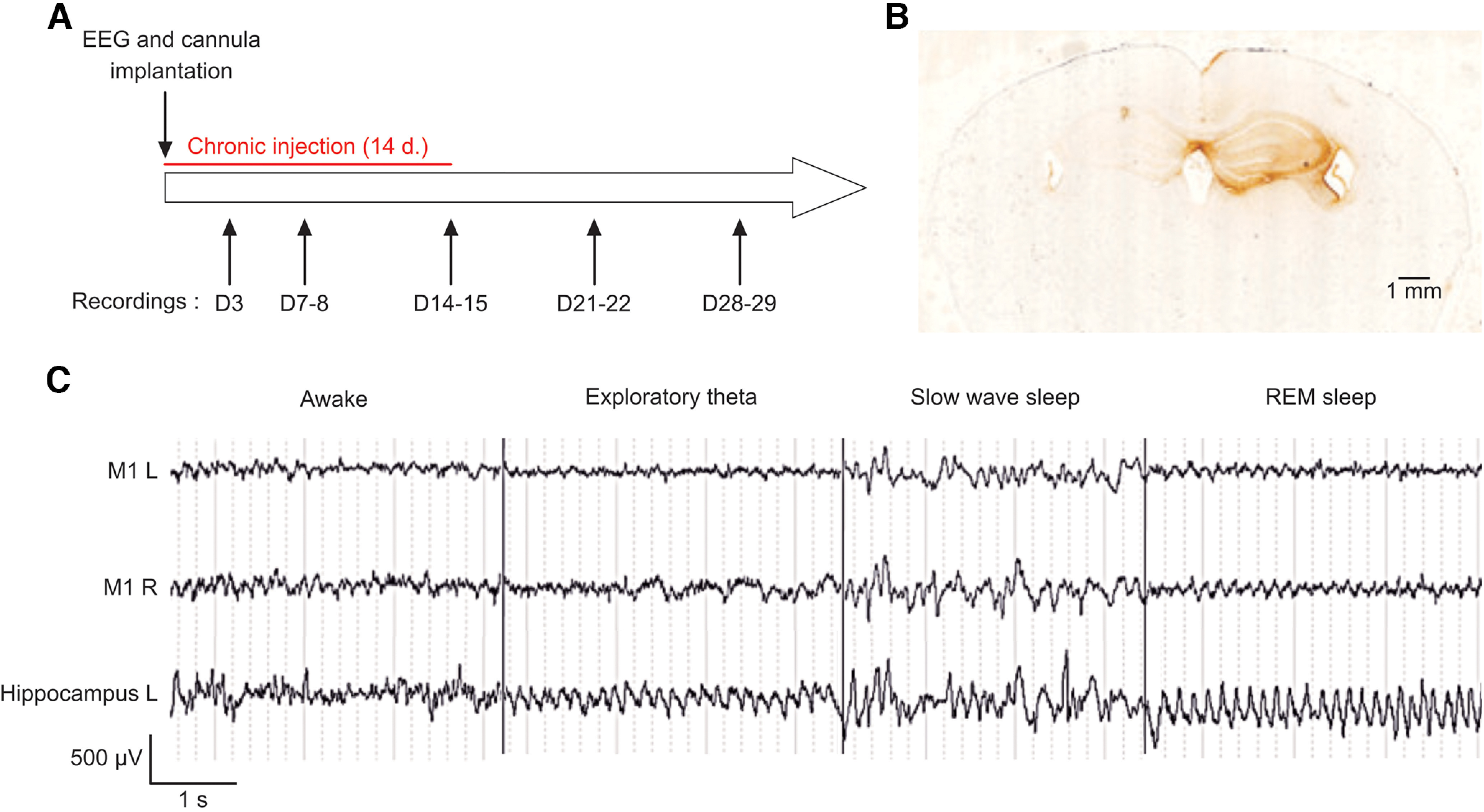
Chronic injections of CSF and purified serum IgG. ***A***, Experimental design. Rats and mice were implanted with an injection cannula in the left hippocampus, in addition to left M1 (M1L) and right M1 (M1R) and bilateral hippocampus electrodes for long-term EEG monitoring of brain activity. The postinjection days of recording are indicated on the schema. ***B***, Representative staining of human IgG on coronal section of a mouse after 7 d of unilateral injection with patient-derived LGI1 serum antibodies to demonstrate the distribution in the hippocampus. Slices were incubated with peroxidase-coupled anti-human IgG followed by DAB staining. ***C***, Typical EEG activity recorded in the different states of vigilance in controls (*n *=* *10 mice and 6 rats) and animals injected with LGI1 antibodies (*n *=* *10 mice; *n *=* *6 rats). Note the absence of epileptiform activity or seizures. Examples of recordings of control and LGI1 awake mice at different time points are shown in Extended Data Figure 2-1. Stereotaxic coordinates used for the chronic injection experiments are reported in Extended Data Table 2-1.

We next investigated whether longer injection, over several days, of anti-LGI1 antibodies could be effective in inducing epileptic seizures. We thus used an osmotic pump connected to an injection cannula implanted in the hippocampus for a continuous release of the antibody-containing solutions (PBS containing purified IgG or CSF) for 2 weeks. The different experimental configurations (*n *=* *36) are listed in Table 3. After 2 weeks of injection, a continuous video-EEG monitoring of the animals was undertaken for another 14 d. Animals were first recorded at post-injection day 3 (D3) for 24 h, and then once a week for 48 h (D7–D8, D14–D15, D21–D22, D28–D29), corresponding to a total of ∼200 h of recordings per animal (Fig. 2*A*). In six animals (*n *=* *3 rats; *n *=* *3 mice), we ensured the diffusion of human antibodies in the hippocampus by immunohistochemical detection of human IgG 7 d after the start of the injection of purified serum IgG (Fig. 2*B*).

10.1523/ENEURO.0267-22.2023.f2-1 Figure 2-1 ***A***, ***B***, Examples of recordings of control (***A***) and LGI1 (***B***) awake mice at different time points: D3, D7, D14, and D30 after injection. Note the absence of epileptiform activity or seizures. M1L, Left motor cortex; M1R, right motor cortex. Download Figure 2-1, TIF file.

10.1523/ENEURO.0267-22.2023.tab2-1 Table 2-1 Coordinates used for injection and electrode placement in the chronic injection experiments. All coordinates are expressed in millimeters from bregma, and were derived and adjusted from the rat and mouse brain atlases of Paxinos and Watson (1997a, b). Download Table 2-1, DOC file.

No animals died during the weeks of injection and the weeks of EEG recording that followed. We did not observe any behavioral differences among the animals of the different groups during handling: no particular aggressiveness, motor hyperactivity, or apathy. EEG activity of control (*n *=* *10 mice; *n* = 6 rats) and LGI1 (*n *=* *14 mice; *n *=* *6 rats) animals was characterized by normal physiological activities during wakefulness (exploratory hippocampal theta waves) and sleep (slow delta waves during phases of deep sleep and theta activity during rapid eye movement (REM) sleep; Gottesmann, 1992; Fig. 2*C*). Sudden and brief twitches of the trunk of the animal were observed in all animals during REM sleep episodes.

We did not detect any epileptic seizures or paroxysmal epileptic abnormalities, such as spikes, polyspikes or spike waves, on the EEGs of control animals or animals injected with anti-LGI1 autoantibodies after exhaustive analysis of the records. Overall, these results show that, like acute injections, chronic injections of CSF or purified total IgG did not induce abnormal epileptic activity that could be recorded on the EEG in both LGI1 and control groups of rats and mice.

## Discussion

Several studies indicated pathogenic effects of anti-LGI1 antibodies through altered expression and function of Kv1 and AMPA receptor (AMPAR) channels (Ohkawa et al., 2013; Petit-Pedrol et al., 2018; Kornau et al., 2020; Ramberger et al., 2020; Extrémet et al., 2022). However, the causal link between these deleterious molecular interactions and seizure activity has never been demonstrated. The effect of anti-LGI1 antibody injections *in vivo* has been previously examined 7 d after hippocampal injection (Ramberger et al., 2020) or after 14 d of chronic infusion into brain ventricles (Petit-Pedrol et al., 2018). However, while these experimental procedures led to a reduced expression of Kv1.1 and AMPAR, as well as an increased excitability of hippocampal neurons, the authors did not use appropriate electrophysiological recordings to assess the presence or the absence of seizures.

We filled this gap by studying the effect of acute or chronic intracerebral infusion of anti-LGI1 autoantibodies, in rats and mice, using multisite electrophysiological recordings allowing direct detection of possible epileptic activity. In the acute injection protocol, we targeted the hippocampus and the M1 cortex, which are the two main regions affected in anti-LGI1 AIE (Navarro et al., 2016). We found that the injection of CSF or IgG purified from serum of anti-LGI1 AIE patients into hippocampus or M1 in rats did not induce epileptiform pattern in both ECoG and LFP. Similar results were obtained with a chronic hippocampal injections protocol. Upon diffusion of human IgG from anti-LGI1 patients, no epileptic abnormalities could be observed on the video-EEG, in mice and in rats chronically injected for 14 d.

The lack of epileptic phenotype could arise from multiple mechanisms. First, as the human disease evolves from several weeks to months, it is possible that the duration of the injection of antibodies was not sufficient to induce seizures and related electrophysiological and molecular defects. Indeed, previous studies reported a 10–15% decrease in Kv1.1 expression after 2 weeks of infusion of anti-LGI1 antibodies (Petit-Pedrol et al., 2018), whereas in LGI1^−/−^ mice, with frequent generalized seizures, Kv1.1 density is decreased by >50% compared with control mice (Seagar et al., 2017). In the most frequent AIE, the anti-NMDA receptor (NMDAR) AIE, patients typically exhibit rapid progression of neuropsychiatric manifestations that can lead to coma within days or weeks (Dalmau et al., 2017). After chronic injection of anti-NMDAR AIE patients’ antibodies into the hippocampi of mice *in vivo*, behavioral changes and memory deficits were observed (Planagumà et al., 2015; Taraschenko et al., 2019), as well as after 6 d of an acute injection (Würdemann et al., 2016). However, no differences in anxiety and locomotion were detected between anti-NMDAR and control animals (Planagumà et al., 2015). Moreover, mice were reported to be either seizure free (Planagumà et al., 2015; Würdemann et al., 2016) or to exhibit nonconvulsive seizures (Taraschenko et al., 2019). Thus, anti-NMDAR antibody injections in mice appears to result in an attenuated phenotype compared with AIE patients. This suggests that the passive antibody transfer method, crucial for demonstrating the pathogenicity of autoantibodies, does not appear suitable for reproducing the epileptic phenotype of AIE patients. In this context, our results, reporting the absence of seizure during acute and chronic injections of anti-LGI1 antibodies, seem consistent with those in the recent literature.

Other factors can explain our negative results. Incubation of anti-LGI1 antibodies on organotypic hippocampal cultures for 3 d decreased the number of GluA1 subunits of AMPAR expressed at the synapse by >50% (Ohkawa et al., 2013). In comparison, after 14 d of *in vivo* injection, GluA1 was only decreased by 10% compared with controls (Planagumà et al., 2015). This could be explained by a slow diffusion of antibodies in the brain, which would greatly reduce the access of autoantibodies to their antigenic targets, in contrast to isolated neuron cultures or brain slices where antibodies can directly access a large neuronal surface. Accordingly, real-time imaging techniques have been used to measure the diffusion properties of nonspecific fluorescently labeled IgG after injection into agarose or into the cortex of adult rats *in vivo* (Wolak et al., 2015). IgG diffusion was shown to be ∼10-fold greater in agarose than in brain. The corresponding diffusion rate of antibodies in the brain was 6.10–8 cm^2^/s, which corresponds to 1 mm^2^ in 41 h. This slow diffusion could arise from the structural properties of the brain microenvironment, but also from specific IgG features, such as their size, shape, and electrical charge. In addition, nonspecific binding of IgG to brain proteins, especially via their Fc fraction, could slow down or stop its diffusion (Wolak et al., 2015). Specific binding, especially in case of autoantibodies that by definition have specific targets, could also slow diffusion. Therefore, the acute injection method followed by 5–10 h of recording may be insufficient to study the effect of autoantibodies on neuronal activities because: (1) this duration would not allow the antibodies to reach a sufficiently large amount of autoantigens; and (2) several days of chronic injection of anti-LGI1 antibodies are needed to affect significantly the amount of Kv1.1 (Petit-Pedrol et al., 2018).

The lack of seizures triggered after chronic 14 d injections, as well as the relatively small decrease in Kv1.1 and AMPAR (Petit-Pedrol et al., 2018), could therefore be because of injection durations that were too short, as it has been shown that longer injection leads to larger decreases of Kv1.1 and AMPAR (Petit-Pedrol et al., 2018). However, the effectiveness of an infusion >14 d may be limited by the instability of the antibodies, which are at ∼37°C in a subcutaneous pump with a half-life time of ∼15–30 d. (Mankarious et al., 1988). The use of a refillable pump could allow the antibody solution to be replenished and prevent the antibodies from remaining at room temperature for too long (Tan et al., 2011). An alternative approach could be the immunization of the animals against the LGI1 protein, so that they produce autoantibodies themselves. Active opening of the blood–brain barrier would also be required to allow antibodies to access the CNS, as has been done in models of experimental allergic encephalomyelitis (Reboldi et al., 2009) or Sydenham’s chorea (Platt et al., 2017).

The lack of seizure induction in the various *in vivo* antibody injection methods we have performed suggests that passive delivery of human antibodies *in vivo* in rodents is not an appropriate method for modeling the epileptic symptoms of AIE. The development of animal models, wherein ion channels downstream to LGI1 will be impaired with specific inhibitory toxins could provide a promising approach to overcome these limitations. An interesting target could be Kv1 channels, whose expression and function are known to be impaired by downregulation of LGI1 (Seagar et al., 2017; Petit-Pedrol et al., 2018; Zhou et al., 2018; Kornau et al., 2020; Lugarà et al., 2020; Ramberger et al., 2020; Extrémet et al., 2022). Indeed, the reduction of Kv1 currents because of the loss of LGI1 function is associated with increases in neuronal excitability and activity, in both genetic and autoimmune models (Baudin et al., 2021), and their pharmacological blockade has been shown to contribute to LGI1-related seizures in a recent animal model (Baudin et al., 2022).

## Synthesis

Reviewing Editor: Christophe Bernard, INSERM & Institut de Neurosciences des Systèmes Decisions are customarily a result of the Reviewing Editor and the peer reviewers coming together and discussing their recommendations until a consensus is reached. When revisions are invited, a fact-based synthesis statement explaining their decision and outlining what is needed to prepare a revision will be listed below. The following reviewer(s) agreed to reveal their identity: Olivier Pascual.

The present work aimed to evaluate the effect of human anti-LGI1 antibodies from patients with autoimmune encephalitis on the emergence of seizures in rodent models. The authors report that anti-LGI1 IgG does not produce acute seizures, somewhat calling into question the possibility that these IgGs may have a causal role in epileptogenesis. Despite these negative results, both reviewers felt that the study was important, that the experiments were carefully conducted, and that the work would be of interest to the community. However, they feel the study may be somewhat preliminary and raised questions about the animal models and, most importantly, the lack of quantitative and statistical analysis of the data.

It is important that the following issues be addressed before proceeding with the review of the manuscript:

1. It is unclear whether human IgG actually recognizes the rodent protein. Immunoprecipitation assays from rat and mouse brain protein extracts would help resolve the important question of whether the lack of apparent effect of human sera reflects a lack of immunogenicity in rodents.

2. It would also be important to estimate the concentration of auto-antibodies in the samples used in this study.

3. The data as presented are primarily qualitative. The authors should endeavor to select some particularly relevant features of the ECoG and LFP (e.g., power, high-frequency oscillations, fast ripples, multiunit activity, bursting...) that may represent characteristics of hyperexcitable networks and perform appropriate statistical analysis on these (including estimation of statistical power, given small Ns).

4. In this context, given that the ECoG recordings were performed in parallel with the LFP recordings, how come the LFP signal was not analyzed? It appears that the data presented in this ms (0-30 Hz) are exclusively from ECoG recordings. Please comment.

## Author Response

Responses to the Editor’s comments:

1. It is unclear whether human IgG actually recognizes the rodent protein. Immunoprecipitation assays from rat and mouse brain protein extracts would help resolve the important question of whether the lack of apparent effect of human sera reflects a lack of immunogenicity in rodents.

The LGI1 protein is highly conserved between human and rodents. The human and rodent proteins have the same number of amino-acids and differ in the nature of only 17/557 amino-acids (3%) (source: www.uniprot.org). None of these 17 amino-acids are involved in the known pathogenic mutations, i.e. the 45 distinct pathogenic mutations of LGI1 identified in the human autosomal dominant epilepsies (Baudin et al., 2021).

Previous studies have shown that the human anti-LGI1 autoantibodies can react with the LGI1 rodent protein, as evidenced by the labeling of human anti-LGI1 IgG on wild-type rodent hippocampal slices, and not on LGI1-/- rodent hippocampal slices (Kornau et al., 2020; Ramberger et al., 2020). Furthermore, injection of human anti-LGI1 autoantibodies into mice brain induced electrophysiological and immunohistological changes (not triggering seizures though), suggesting that they were able to interact with the mouse protein (Extrémet et al., 2022; Petit-Pedrol et al., 2018; Ramberger et al., 2020)

To clarify this issue, we have added a sentence in the 2nd paragraph of the introduction (p. 3):

“LGI1 is highly conserved between human and rodents. Previous studies reported a specific labeling of human anti-LGI1 IgG on rodent wild-type hippocampal slices, suggesting that the human autoantibodies effectively react with the rodent protein (Extrémet et al., 2022; Kornau et al., 2020; Ramberger et al., 2020).”

2. It would also be important to estimate the concentration of auto-antibodies in the samples used in this study.

We performed a semi-quantitative estimation of anti-LGI1 antibodies in the IgG purified from serum samples, through titration and labeling on HEK293 cells expressing LGI1 (FA1439-1003-1, EuroImmun). We diluted the IgG sample progressively by adding PBS such as the volume is multiplied by two between each staining. We reported the titration ratio for each sample in the Table 1, which corresponds to the last dilution in which we were able to detect a staining on HEK cells. We could not perform this analysis on CSF sample because of the difficulty to obtain CSF and the small volume collected that prevented the titration to be done. Importantly, a positive research for anti-LGI1 autoantibodies was systematically performed before the use of CSF.

To follow the Referee’s comment, these precisions are now added to the Material and methods section (paragraph “Purification of IgG from patient serum”) (p.5):

“Semi-quantitative estimation of anti-LGI1 antibodies was performed on the IgG purified from serum samples, through titration and labeling on HEK293 cells expressing LGI1 (FA1439-1003-1, EuroImmun). We diluted the IgG sample progressively by adding PBS such as the volume was multiplied by two between each staining. The titration ratio for each sample is reported in Table 1 and corresponds to the last dilution in which we could detect a staining on HEK cells.”

We also modified the legend of Table 1 accordingly (p.24):

“The serum anti-LGI1 Ab titration ratio corresponds to the last dilution of the samples in which a staining on HEK cells was detected.”

3. The data as presented are primarily qualitative. The authors should endeavor to select some particularly relevant features of the ECoG and LFP (e.g., power, high-frequency oscillations, fast ripples, multiunit activity, bursting...) that may represent characteristics of hyperexcitable networks and perform appropriate statistical analysis on these (including estimation of statistical power, given small Ns).

The thorough and systematic screening of all our recordings did not reveal any epileptic seizures, nor particular events such as surges of high-frequency oscillations or fast ripples. We did perform a comparative analysis of the frequency content of electrical signals before and after injection of autoantibodies in the acute experiments where LFP activity was acquired from multiple locations relative to the injection site. This analysis confirmed the lack of significant differences between LG1 and control conditions (Fig. 1).

Following the Referee’s suggestion, we have now added precisions concerning the estimation of the statistical power:

-in the Material and Methods section (paragraph “Analysis of electrophysiological signals”) (p.9): “The statistical power was estimated for each frequency bin as the probability to find a difference of {plus minus} 50% knowing the mean and SD control values, using the “sampsizepwr” built-in MATLAB function.”

-in the legend of the Figure 1D (p.22): “No significant difference was found between Control (M1, n = 8; Hippocampus, n = 8) and LGI1 (M1, n = 14; Hippocampus, n = 12) experiments (two-tailed Mann-Whitney rank-sum test for each time period, on data binned in frequency bands of 1 Hz width). The statistical power estimated for each frequency bin was on average 0.55 {plus minus} 0.15.”

4. In this context, given that the ECoG recordings were performed in parallel with the LFP recordings, how come the LFP signal was not analyzed? It appears that the data presented in this ms (0-30 Hz) are exclusively from ECoG recordings. Please comment.

As illustrated in Figure 1, the frequency content of LFP (and not EcoG) signals were analyzed in acute experiments, focusing on the recording site closest to the injection site. This analysis is described in the results section (page 10).

Responses to Reviewer #1 comments

1. There is no question regarding the quality of experiments and analysis being made, but as some epilepsies and autoimmune limbic encephalitis in particular are diseases that deeply rely on the genetic background. The major issue concerns the experimental conditions used for the demonstration, as one may wonder if the authors used the best combination of experimental conditions to favor seizures? Is the C57BL6J strain the best mouse strain to induce epilepsy as this strain was found to be less prone to epilepsies than balb/c or FVB/NJ strain for example? doi: 10.1016/j.neulet.2011.09.030, 10.1016/j.yebeh.2019.106547. The same matter holds true for SD rats DOI: 10.1016/j.yebeh.2016.05.020, this should be discussed.

We agree with the Referee that it could also have been interesting to consider other rodent strains. However, our choice was motivated by the fact that C57BL6J mice were used in previous studies of in vivo intracerebral injections of anti-LGI1 human auto-antibodies, showing that anti-LGI1 auto-antibodies do induce electrophysiological and immunohistological disturbances in this mouse strain (Kornau et al., 2020; Petit-Pedrol et al., 2018; Ramberger et al., 2020). We thus selected this mouse strain to be able to confront and interpret our results in the light of previous findings. The SD rats were chosen as it is the strain we used to develop our LGI1 encephalitis seizure-like model, which recapitulates patients’ EEG and behavioral seizures after intracerebral injection of dendrotoxin-K (Baudin et al., 2022).

We now justified the choice of the strains in the Material and Methods “Animals” paragraph (p.5):

“The C57BL6J mouse strain was chosen as it is commonly used in previous in vitro and in vivo studies addressing the effects of anti-LGI1 human auto-antibodies (Kornau et al., 2020; Petit-Pedrol et al., 2018; Ramberger et al., 2020). The Sprague Dawley rat strain is the one used to develop our LGI1 encephalitis seizure-like model, which recapitulates the electrical and behavioral hallmarks of patients (Baudin et al., 2022).”

2. The design of experiment is also questionable, as there are multiple conditions for very few n. This led the authors to pool their results for statistical reasons, I believe. This choice is questionable regarding the previous issues raised on the genetic background and given the fact that CSF and serum might have different properties. Although splitting the results would not change the overall message of the study, this could add some degree of precision. Maybe the authors could add in supplemental data the analysis for the groups mice csf, mice serum, rats csf and rats serum if available so that the reader can appreciate the individual results.

As pointed by the Referee, we indeed pooled our results for statistical reasons. To follow his/her suggestion, we have now added a new figure (Extended Data Fig. 1-1) illustrating the same results as those displayed in the Fig.1, but separated between CSF and serum data. Those experiments were only performed on rats.

The Results section has been modified as follows (p.10): “The frequency content of LFP activities after injection was similar between Control and LGI1 experiments, when all experiments were pooled together (Fig. 1C and 1D) as well as when serum IgG experiments and CSF experiments were analyzed separately (Extended data Fig. 1-1).”

Responses to the Reviewer #2 comments

1. The variability of age and antecedents of mesial temporal seizures from patients could provide a bias in the analysis. Please comment.

Differences in anti-LGI1 autoantibodies expression level between patients, or in the domains of the protein targeted by the autoantibodies, can be involved in the variety of symptoms described in anti-LGI1 encephalitis (i.e. patients with dominant mesial temporal lobe seizures, or patients with dominant motor seizures (Baudin et al., 2021)).

Patients with different profiles were thus selected (different ages, with or without mesial temporal seizures, with more or less frequent motor seizures, etc.) to increase our chance to trigger seizures because it was not possible to identify a priori the most pro-epileptic profile. This approach is indeed questionable. However, none of our injection was able to induce seizure, regardless of the patient profile. We thus think that pooling all the data together does not represent a bias. We certainly would have presented the results differently if different effects had been observed.

Those precisions have been added to the Material and Methods (paragraph “Patients”) (p.4): “Samples from different patients were used independently in order to be able to assess any specific patient-dependent effect and to avoid drawing general conclusions from effects caused by a specific patient profile.”

2. Robust clinical and electrical analysis of seizure should be described in details; recording from control and LGI1 should be provided at different time points as well.

To follow the pertinent remark of the Reviewer, we added a new figure (Extended Data Fig. 2-1) illustrating examples of recordings of LGI1 and control mice at different time points: D3, D7, D14 and D30 after injection. Seizure analysis could not be performed because our thorough and systematic screening of the data did not permit to detect any seizures. The criteria used to detect seizures are described in the Materiel and Methods was as follows: ‘an abrupt onset and termination, an amplitude threshold clearly different from the baseline activity (at least 3 times the SD), and/or abnormal changes in the background rhythm. Myoclonus and other epileptic movements, whether or not associated with events on the EEG, were also looked for on video recordings.

3. Rationale/justification for the use or rat or mice including unilateral injection in hippocampus or cortex should be provide and develop more in depth.

The following information is now provided in the manuscript:

-Material and Methods, paragraph “Animals” (p.5): “Both rat and mice were used to verify that the absence of seizure was not species specific.”

-Material and methods, paragraph “Acute injections and multi-scale in vivo recordings in sedated rats” (p.6): “Unilateral injections in the hippocampus or cortex were preferred to bilateral injections to avoid traumatizing large brain volumes and to offer the possibility to detect focal seizures and determine their initiation site.”

4. IRB approval could be required.

We added in the Material and Methods, “Patients” paragraph (p.4):

“The protocol was sponsored by INSERM (C16-16, 20152482) and approved by a local ethics committee.”
