# Supplementary figures and images for: In Vivo Injection of Anti-LGI1 Antibodies into the Rodent M1 Cortex and Hippocampus Is Ineffective in Inducing Seizures
Source: eNeuro. 2023 Mar 13;10(3):ENEURO.0267-22.2023. doi: 10.1523/ENEURO.0267-22.2023 (PMC10012326; doi:10.1523/ENEURO.0267-22.2023)

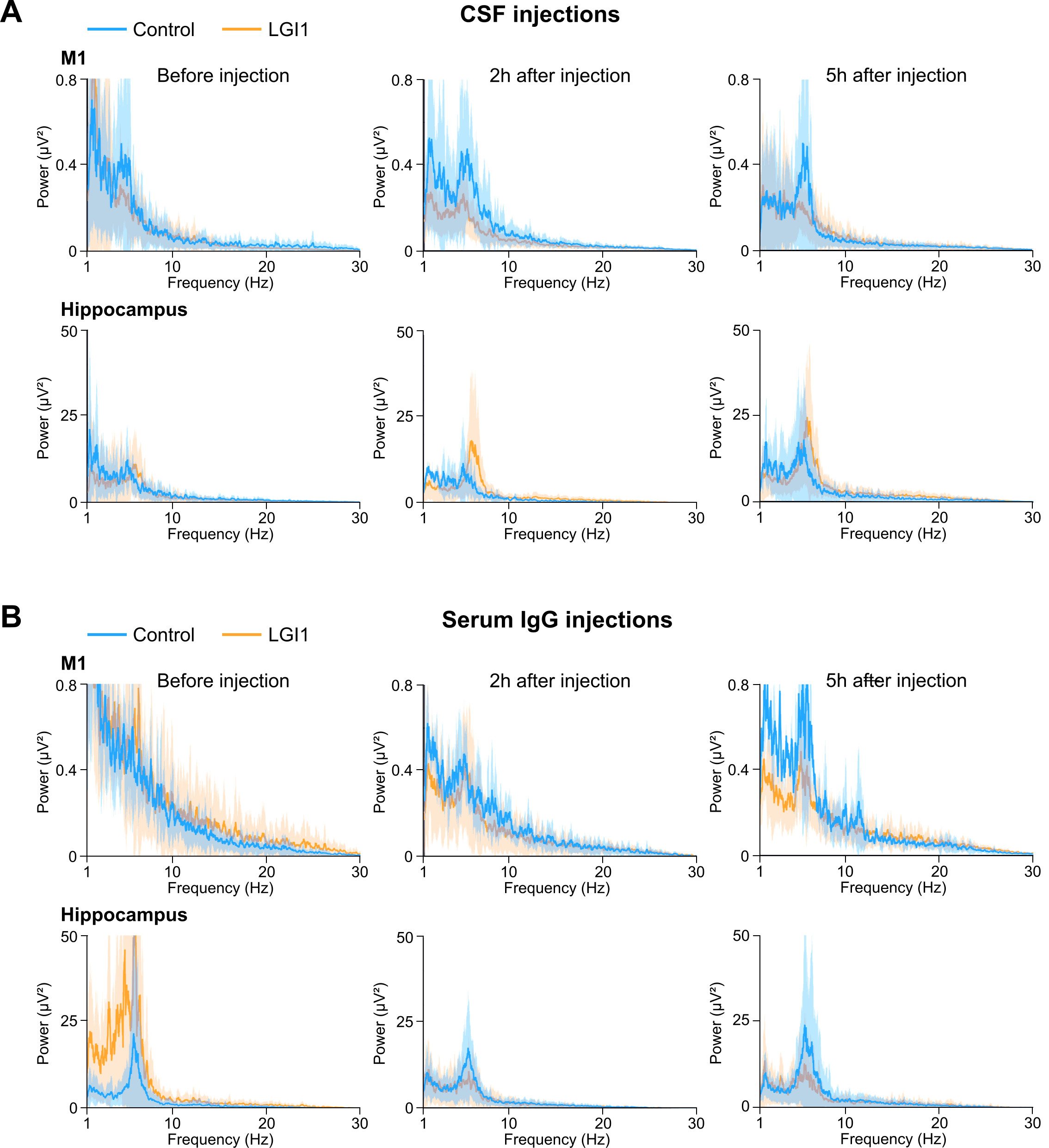

Supplement: Figure 1-1 — A, B, Frequency content of LFP activities after acute injection of CSF (A) and serum IgG (B) from LGI1 and control patients in rat. A, Frequency power (mean ± SD) of intracerebral LFP recordings after CSF injection in M1 (top) and hippocampus (bottom), in control (blue) and LGI1 (orange) conditions. Electrophysiological signals were analyzed before injection (left), 2 h after injection (middle), and 5 h after injection (right). The electrode closest to the injection site was selected for the analysis. No significant difference was found between control (M1, n = 5; hippocampus, n = 5) and LGI1 (M1, n = 8; hippocampus, n = 6) experiments (two-tailed Mann–Whitney rank-sum test for each time period, on data binned in frequency bands of 1 Hz width). B, Same analysis as the one presented in A, but after serum IgG injection. No significant difference was found between control (M1, n = 3; hippocampus, n = 3) and LGI1 (M1, n = 6; hippocampus, n = 6) experiments (two-tailed Mann–Whitney rank-sum test for each time period, on data binned in frequency bands of 1 Hz width). Download Figure 1-1, TIF file. [file enu-eN-NRS-0267-22-s02.tif]

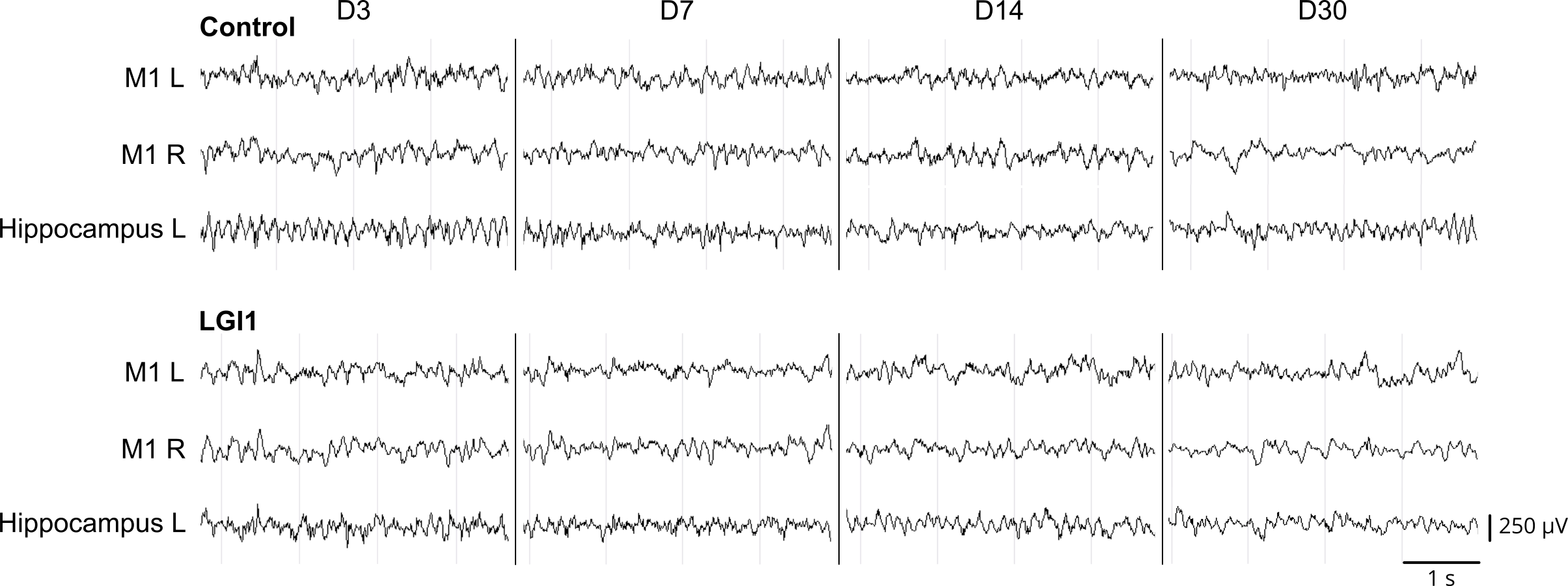

Supplement: Figure 2-1 — A, B, Examples of recordings of control (A) and LGI1 (B) awake mice at different time points: D3, D7, D14, and D30 after injection. Note the absence of epileptiform activity or seizures. M1L, Left motor cortex; M1R, right motor cortex. Download Figure 2-1, TIF file. [file enu-eN-NRS-0267-22-s03.tif]
