# Supplementary material for: In Vivo Injection of Anti-LGI1 Antibodies into the Rodent M1 Cortex and Hippocampus Is Ineffective in Inducing Seizures
Source: eNeuro. 2023 Mar 13;10(3):ENEURO.0267-22.2023. doi: 10.1523/ENEURO.0267-22.2023 (PMC10012326; doi:10.1523/ENEURO.0267-22.2023)
Supplement: Table 1-1 — Coordinates used for injection and electrode placement in the acute injection experiments. All coordinates are expressed in millimeters from bregma, and were derived and adjusted from the rat brain atlas of Paxinos and Watson (1997a). Download Table 1-1, DOC file. [file enu-eN-NRS-0267-22-s04.doc]

| **Hippocampal injection experiments** | | | |
| --- | --- | --- | --- |
| **Brain structure** | **Antero-posterior** | **Medio-lateral** | **Depth** |
| Right and left M1 cortex: ECoG | 3 | +/-2.6 | - |
| Left APC: ECoG | -3.6 | 3.5 | - |
| Left hippocampus: multichannel probe | -3.4 | 2.5 | Between 0 and -3.75 (16 electrodes) |
| Left hippocampus: injection site | -3.6 | 2.5 | -3 |
| **M1 cortex injection experiments** | | | |
| **Brain structure** | **Antero-posterior** | **Medio-lateral** | **Depth** |
| Left M1 cortex: multichannel probe | 3 | 2.6 | Between 0 and -3.75 (16 electrodes) |
| Left M1 cortex: injection site | 2.6 | 2.6 | -1 |
| Right M1 cortex: ECoG | 3 | -2.6 | - |
| Left APC: ECoG | -3.6 | 3.5 | - |
