# Supplementary material for: In Vivo Injection of Anti-LGI1 Antibodies into the Rodent M1 Cortex and Hippocampus Is Ineffective in Inducing Seizures
Source: eNeuro. 2023 Mar 13;10(3):ENEURO.0267-22.2023. doi: 10.1523/ENEURO.0267-22.2023 (PMC10012326; doi:10.1523/ENEURO.0267-22.2023)
Supplement: Table 2-1 — Coordinates used for injection and electrode placement in the chronic injection experiments. All coordinates are expressed in millimeters from bregma, and were derived and adjusted from the rat and mouse brain atlases of Paxinos and Watson (1997a, b). Download Table 2-1, DOC file. [file enu-eN-NRS-0267-22-s05.doc]

| **Rat experiments** | | | |
| --- | --- | --- | --- |
| **Brain structure** | **Antero-posterior** | **Medio-lateral** | **Depth** |
| Right and left M1 cortex: EEG | 3 | +/-2.6 | - |
| Left anterior hippocampus: chronic injection cannula | -2 | 1.5 | -3 |
| Left posterior hippocampus: intracranial electrode | -3.4 | 2.5 | -3 |
| Cerebellum: reference | -10 | 0 | - |
| **Mice experiments** | | | |
| **Brain structure** | **Antero-posterior** | **Medio-lateral** | **Depth** |
| Right and left M1 cortex: EEG | 1.8 | -1.8 | - |
| Left anterior hippocampus: chronic injection cannula | -1 | -0.4 | -2.3 |
| Left posterior hippocampus: intracranial electrode | -1.8 | -1.8 | -2.3 |
| Cerebellum: reference | -6.5 | 0 | - |
